# Real-Time Monitoring Platform for Ocular Drug Delivery

**DOI:** 10.3390/pharmaceutics15051444

**Published:** 2023-05-09

**Authors:** Sahar Awwad, Nkiruka Ibeanu, Tianyang Liu, Angeliki Velentza-Almpani, Nerisha Chouhan, Stavros Vlatakis, Peng Tee Khaw, Steve Brocchini, Yann Bouremel

**Affiliations:** 1 Optceutics Ltd., 28a Menelik Road, London NW2 3RP, UK; nkiruka.ibeanu.17@ucl.ac.uk (N.I.); a.almpani@ucl.ac.uk (A.V.-A.); nerisha.chouhan.19@ucl.ac.uk (N.C.); stavros.1.vlatakis@kcl.ac.uk (S.V.); p.khaw@ucl.ac.uk (P.T.K.); s.brocchini@ucl.ac.uk (S.B.); 2 UCL School of Pharmacy, 29-39 Brunswick Square, London WC1N 1AX, UK; 3 National Institute for Health Research (NIHR) Biomedical Research Centre, Moorfields Eye Hospital NHS Foundation Trust and UCL Institute of Ophthalmology, London EC1V 9EL, UK

**Keywords:** ocular, ophthalmology, automation, real-time monitoring, concentration probes, saccades, microfluidics, pharmaceutical testing

## Abstract

Real-time measurement is important in modern dissolution testing to aid in parallel drug characterisation and quality control (QC). The development of a real-time monitoring platform (microfluidic system, a novel eye movement platform with temperature sensors and accelerometers and a concentration probe setup) in conjunction with an in vitro model of the human eye (PK-Eye™) is reported. The importance of surface membrane permeability when modelling the PK-Eye™ was determined with a “pursing model” (a simplified setup of the hyaloid membrane). Parallel microfluidic control of PK-Eye™ models from a single source of pressure was performed with a ratio of 1:6 (pressure source:models) demonstrating scalability and reproducibility of pressure-flow data. Pore size and exposed surface area helped obtain a physiological range of intraocular pressure (IOP) within the models, demonstrating the need to reproduce in vitro dimensions as closely as possible to the real eye. Variation of aqueous humour flow rate throughout the day was demonstrated with a developed circadian rhythm program. Capabilities of different eye movements were programmed and achieved with an in-house eye movement platform. A concentration probe recorded the real-time concentration monitoring of injected albumin-conjugated Alexa Fluor 488 (Alexa albumin), which displayed constant release profiles. These results demonstrate the possibility of real-time monitoring of a pharmaceutical model for preclinical testing of ocular formulations.

## 1. Introduction

Back of the eye or posterior segment diseases (e.g., age-related macular degeneration (AMD), glaucoma and diabetic retinopathy) are major blinding diseases that affect millions worldwide and are becoming more prevalent with an increase in the ageing population [1]. Therapeutic antibodies and proteins have revolutionised the treatment of chronic blinding conditions that impact our ageing population. Most intraocular medicines are frequently administered via direct injections (i.e., intravitreal injections) to achieve high and reproducible doses in the posterior segment. However, repeated intravitreal administration is understandably difficult for patients and healthcare systems, and poses potential risks [2]. The emergence of new therapeutic entities in the market and the need to address this unmet healthcare challenge have created a need for the continued application of formulation strategies to increase or prolong the duration of the action of drugs to treat ocular diseases [3].

New formulations and dosing regimens are evaluated during preclinical development in animal models. Animal models play an important role in the Research & Development (R&D) of drug delivery formulations for all routes of administration. Obtaining ocular pharmacokinetic data is an intrusive/invasive procedure, where a needle is sometimes inserted directly into the animal eye to aspirate fluid at various time points, or an animal is sacrificed at selected time-points after drug administration. Some limitations of frequently used animal models for ocular testing include obvious anatomical and aqueous outflow differences between animal and human eyes that can affect estimation of ocular pharmacokinetics [4]. Another challenge that is observed during the testing of biologics is the formation of anti-drug antibodies (ADAs) that usually occurs in response to a human protein and is an intractable problem for the industry. Ocular tolerability, e.g., inflammation, increase in intraocular pressure (IOP) and endophthalmitis, raises concerns during formulation testing [5]. High costs of animal models, including ethical considerations and limitations, are additional issues related to the use of animal models for evaluating drug pharmacokinetics. It is crucial to have other testing techniques beyond animal models to facilitate preclinical development. Alternative methods can also help save costs and cut years from the R&D paths for ocular formulations.

Standardised in vitro methods are crucial requirements described in the compendia for quality control (QC) testing and for formulation characterisation (e.g., dissolution and pharmacokinetic analyses) [6,7]. In vitro models can be used as an alternative or a supplement to the use of animal models during preclinical testing. A wide range of in vitro models have been reported in the pharmacopeia [8,9,10,11,12] for different routes of administration, e.g., pulmonary, percutaneous and oral routes [13,14,15,16]. These models can help reduce the number of animal studies during formulation development and can help validate the use of dissolution models to establish dissolution specifications [17]. The preclinical development and optimisation of formulations is crucial for most new medicines. In vitro studies can help in demonstrating bioequivalence of an active pharmaceutical ingredient (API) and indication, and understanding of drug behavior [18]. Regulatory guidance for immediate and controlled release formulations has been developed by the US Food Drug and Administration (FDA) to reduce the number of bioavailability studies required to demonstrate bioequivalence [17]. While there has been tremendous progress with in vitro dissolution testing for other routes of administration, there is no approved or official model reported in the pharmacopeia specially designed to determine the intraocular pharmacokinetics of drugs. Apparatus IV (flow-through cell) and Apparatus VII (reciprocating holder) are the apparatuses described in the pharmacopeia that have been used to study drug release from extended-release dosage forms, mainly for oral administration. Apparatus VII has also been adapted for testing intravitreal products, in particular Ozurdex^®^. However, these models do not effectively model the eye with regards to flow rate, viscosity and compartmentalisation, which are important parameters in understanding drug kinetics and stability in the eye.

**Figure 1:**
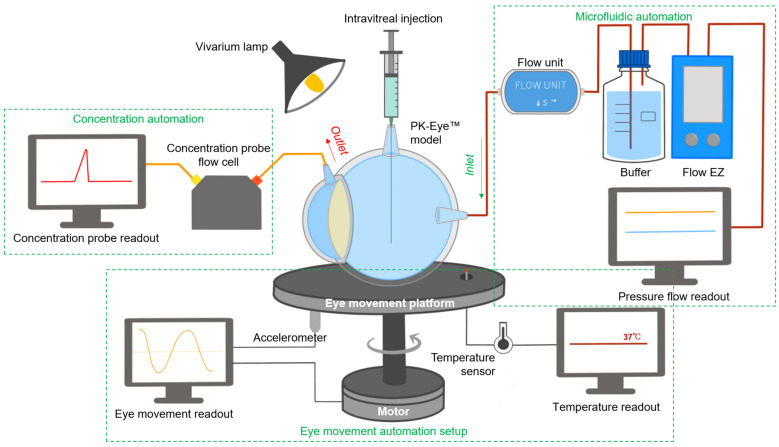
Schematic of the real-time monitoring platform of the PK-Eye™ model. The platform setup included a microfluidic automation system for flow analysis, an eye movement automation platform for investigating the effects of eye movement and a concentration automation setup (with the use of probes) for clearance estimation of labelled proteins. All these setups were used in parallel to provide constant monitoring of different parameters in ophthalmic drug delivery and development.

In recent years, there has been progress in designing and reporting ocular in vitro models for preclinical testing in research. In vitro models have been designed to evaluate protein stability in simulated vitreous fluids (SVF) [19], to understand the effects of eye movements (EyeMos system) [20,21], and to evaluate the effect of SVF and aqueous outflow on drug release/clearance times (PK-Eye™ models) [22,23,24,25,26,27,28]. PK-Eye™ model variants™ have also been reported in the literature [29,30]. The PK-Eye™ is a robust and simple-to-use in vitro preclinical model that addresses the inherent limitations of in vivo models used to develop long-acting intraocular medicines. Various PK-Eye™ prototypes have been designed over the years to predict mass transfer and have been used for different testing purposes. These include the first-generation PK-Eye™ model [22], posterior inflow model [25], ciliary inflow model [25], retina-choroid-sclera (RCS) model [25] and an intracameral prototype [26]. The effects of diffusion and convection on drug clearance after drug administration from both the back and front of the eye have been previously investigated by varying port placement, and by altering membrane dimensions and properties. These PK-Eye™ prototypes have been used and described in this report in conjunction with a newly designed real-time monitoring platform (Figure 1).

Combination of in vitro models with real-time measurements can help facilitate preclinical testing. Real-time measurement has been reported as one important trend in modern dissolution testing to conduct parallel studies in a cost-effective and efficient manner, which can aid in increasing the amount of collectable data with less manual intervention, resulting in a more robust system. An automated system would allow for real-time monitoring with high-temporal resolution and more accurate pharmaceutical testing [31]; for example, drug release profiles have been optimised and reported using carbon nanodots [32] or optical fibres [33]. The real-time monitoring platform described in this manuscript (Figure 1) was scaled and automated so a large number of experiments could be conducted simultaneously to optimise preclinical candidates. The platform was aimed at providing the proof-of-concept for a fully automated (flow and data acquisition) and optimised PK-Eye™ model testing setup to accelerate intraocular drug development, in the hope of reducing manual, labor-intensive sample analysis while improving accuracy, in order to further accelerate development timelines. The platform introduced monitoring of flow via a microfluidic system, temperature and eye movements via a bespoken programmed eye movement platform, and concentration readout of labelled protein molecules via optic flow cells.

## 2. Materials and Methods

### 2.1. Materials

**Figure 2:**
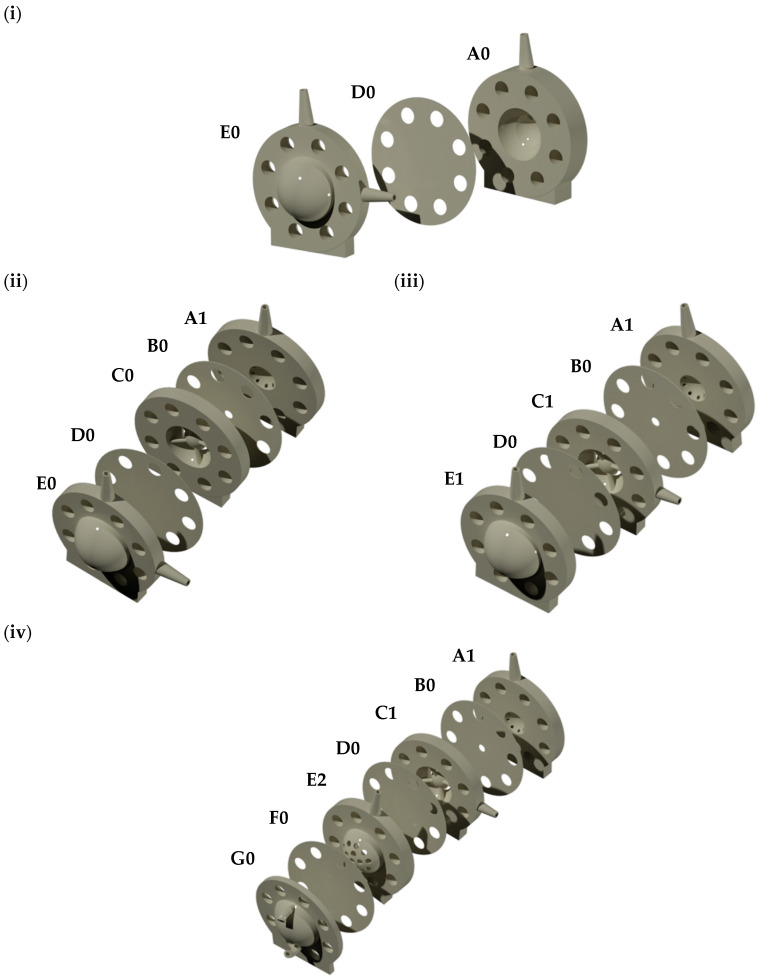
(**i**) First-generation PK-Eye™ model, (**ii**) posterior inflow model, (**iii**) ciliary inflow model and (**iv**) RCS model. **A0:** First generation PK-Eye™ anterior part outlet, **A1:** anterior part outlet (front of the model sampling), **B0:** iris part, **C0:** lens part, **C1:** lens part with aqueous humour inlet, **D0:** hyaloid membrane, **E0:** posterior part with aqueous humour inlet (inflow) and injection port (drug administration), **E1:** posterior part with injection port, **E2:** posterior part for RCS model with injection port (drug administration), **F0:** RCS membrane and **G0:** RCS outlet (back of the model sampling).

Different Visking dialysis membrane tubings (molecular weight cut-off, MWCO 12–300 kDa) were obtained from Medicell International Ltd. (London, UK) and VWR International Ltd. (Leicestershire, UK). Sodium hyaluronate (HA, 1.5–1.8 MDa) was purchased from Lifecore Biomedical, LLC (Chaska, MN, USA). Albumin from bovine serum (BSA) Alexa Fluor 488 (Alexa albumin) was purchased from Life Technologies Ltd. (Paisley, UK). FLPG Plus, LineUp Flow EZ, LineUp LINK module, remote control system, flow units S PACKAGE bundle, LineUP SUPPLY kit, FEP tubing (1/16-254), LineUP ADAPT module, 2-SWITCH, M-SWITCH, and SWITCHBOARD were purchased from Fluigent (Le Kremlin-Bicêtre, France). Form 3B complete package, Formlabs BioMed clear resin, Formlabs tough 2000 resin, Form 3 resin tank v2.1 and Form 3 build platform were purchased from Additive-X Ltd. (Ripon, UK). PIMag^®^ Rotation stage, Ø 32 mm clear aperture, iron core 3-phase torque motor, incremental angle measuring system with sin/cos signal transmission, PIMag^®^ motion controller for magnetic direct drives and extension cable for motor signals and MS D-Sub 15 (f) PI to D-Sub 9 (f) PIM 3m were purchased from Physik Instrumente UK Ltd. (Bedford, UK). The accelerometers PCB IMIB01, the Compact Rio cRIO-9048, 1.3 GHz Dual-Core, 160T FPGA, 8-Slot, RT, XT, NI 9940 Backshell for 36-pos Spring Terminal Connector, NI 9401 8-channel, 100 ns, TTL digital input/output module, NI 9870 4-Port RS 232 serial module W/4 10P10C-DE9 cables, NI 9226 Spring, 8-channel RTD, PT1000, 24-bit, 50S/s/ch AI module, NI 9230 BNC, 3-Ch, ± 30 V, 12.8 kS/s/ch, 24-Bit, IEPE AI Module, NI-9977 C series filler module for empty slot and NI 9924 front-mount terminal block for 25-pin D-sub modules were acquired from NI Corporation Ltd. (Berkshire, UK). The temperature sensors PT-100 were acquired from RS PRO (Corby, UK). QEPRO-ABS thermoelectrically cooled, high sensitivity spectrometer, INTSMA-200 interchangeable slit, LDC-1 single channel LED controller, LSM-470A VIS led module, QP600-1-UV-VIS optical fibres, FIA-SMA-FL-ULT flow cell, OCPLUS-QE-1 and OCEANVIEW software were purchased from Ocean Insight (Florida, USA). Four PK-Eye™ prototypes (Figure 2) were chosen to demonstrate the capabilities of the real-time monitoring platform as proof-of-concept, i.e., the first-generation PK-Eye™ model (Figure 2i), posterior inflow model (Figure 2ii), ciliary inflow model (Figure 2iii) and RCS model (Figure 2iv).

### 2.2. Methods

#### 2.2.1. Pursing Experiments with the Microfluidic System

Simplified single-entry port models (pursing models [34]) were used to investigate the permeability of different membranes for applications in the PK-Eye™. Pursing experiments were conducted to help choose the right hyaloid membrane part to separate the anterior and posterior parts of the model (posterior flow model was used as a proof-of-concept), and to determine the right membrane diameter size and pore size. The pursing models consisted of two parts i.e., a laser-cut top acrylic layer of different surface areas (5.0, 15.0 and 20.0 mm) and a bottom layer (with a single flow inlet and central flow outlet) 3D-printed with BioMed clear resin (a USP class VI certified material for biocompatible applications using the 3D printer Form 3B). The bottom layer was printed with no internal support to ensure its smoothness on both sides. Each model was assembled with a membrane between the two layers and clamped together with 4 tightened screws. The models were then connected to a 69-mbar, 345-mbar or 2-bar Flow EZ pump for membranes with MWCOs of 300 kDa, 50 kDa and 12–14 kDa respectively. The setup consisted of PBS, pH 7.4 buffer, a flow unit connected to the pressure pump, and 254 µm tubing for the connection. The microfluidic system was connected to the computer for pressure control through the Fluigent All-in-One (A-i-O) program and for running an algorithm to automatically increase pressure in a stepwise manner with the Microfluidics Automation Tool (MAT) program. The algorithm was allowed to run for 50 to 60 h, and pressure-flow data were recorded. Interstitial flow rate and pressure were also recorded with these different setups.

#### 2.2.2. 3D Printing of PK-Eye™ Prototypes

All models were printed using the BioMed clear resin. Each part was printed with no internal support to ensure the smoothness of the model and good sealing between the parts. The models were assembled with the help of M6 screws. The anterior cavity was filled with PBS (pH 7.4 and 0.05% sodium azide), whereas the posterior cavity was filled with either PBS or with SVF (3.0 mg/mL HA, ~0.6–0.8 Pa·s). All models were connected to the microfluidic system using PEEK red tubing (external diameter 1.6 mm and internal diameter 127 µm) and PEEK blue tubing (external diameter 1.6 mm and internal diameter 250 µm).

#### 2.2.3. Real-Time Monitoring Platform of PK-Eye™

##### Automating Flow with Microfluidics

1. Scale up capabilities of the microfluidic system

Each PK-Eye™ model was connected to a 345-mbar LineUp Flow EZ microfluidic controller. The setup of the PK-Eye™ model with the LineUp Flow EZ microfluidic controller consisted of buffer (PBS, pH 7.4), a flow unit, and 127 and 254 µm tubing for the flow connection. All the LineUp Flow EZ microfluidic controllers were connected to the FLPG plus 2-bar pump, which was the main pressure source. The microfluidic system was connected to the computer for pressure and flow control through the Fluigent A-i-O program. The models were connected to the microfluidic setup and the experiments conducted to demonstrate scalability were (i) 6 × LineUp Flow EZ connected to 6 × flow sensors and 6 × PK-Eye™ models running simultaneously and (ii) 1× LineUp Flow EZ connected to 1, 2, 3, 4, 5 and 6 flow rate sensors and PK-Eye™ models, to show that 1 microfluidic controller can control 1–6 models, respectively. All experiments were conducted at a fixed flow rate of 2.0 μL/min at 37°C. The microfluidic system was equilibrated for 48 h before data recording.

2. Circadian rhythm using the microfluidic system

The capabilities of the microfluidic system with the model setup were further investigated for diurnal flow rate variations. Physiological aqueous flow rate was programmed with the microfluidic system to demonstrate human sleep–wake states, denoted as circadian rhythm (1.5–3.0 µL/min). The circadian rhythm setup consisted of a LineUp Flow EZ connected to the FLPG pressure generator and a flow rate sensor monitoring the aqueous humor flow rate through a PK-Eye™ model. The circadian program was written using the Fluigent OxyGEN software as a sinusoidal wave to program the flow rate between 1.5 and 3.0 µL/min over a period of 24 h.

3. Demonstrating QC with microfluidic setup

The microfluidic setup can help demonstrate any SVF leakage from the posterior cavity during the testing periods in the hope of maintaining batch-to-batch reproducibility and to ensure more accurate representation of drug clearance. A fresh batch of SVF [25] was prepared and transferred to the posterior cavity of the RCS model. A flow rate of 2.0 µL/min was introduced via the microfluidic system and pressure-flow data were monitored for 5 days to observe any spike in pressure and any alteration to flow.

##### Automating Eye Movement with a Novel Eye Movement Platform

1. Building the eye movement platform

The 3D-printed eye movement platform was screwed to a V-611 rotation stage (Physik Instrumente Ltd. UK, Cranfield, UK) with a high load capacity to hold the platform and the PK-Eye™ models to generate high velocities, and accelerations to mimic the chosen eye movement. The stage has a very high resolution in order to reproduce the micro-saccades. The V-611 is an electromagnetic direct drive controlled with the C-891 PIMag^®^ motion controller (Physik Instrumente Ltd. UK, Cranfield, UK) that enables a very accurate rotation of the stage. The bidirectional repeatability is ±1.5 µrad. The resolution is 0.0001° with a maximum velocity of 3000°/sec and a maximum load of 10 kg-force. Three eye movements that are similar to human eye movements were investigated with the eye movement platform, i.e., slow pursuit, micro-saccadic and saccadic movement.

2. Programming the eye movements

Smooth pursuit settings were the following: 20° in 1.8 s reaching a maximum velocity of 22°/s, stop for 50 ms (velocity of 0°/s), and moving back 20° at the same velocity to reach an angle of 0°. The model then moved back in the opposite direction with the same velocity and angle patterns. Micro-saccadic settings had a rotation of approximately 0.4° that moved back and forth every 1.25 s. The velocity reached a maximum of 25 to 26°/s to cover 0.4° in 20 ms. The saccadic movement had a setting of 2° for movement in 100 ms every 330 ms up to 8° before going back to 0° in 400 ms. The velocity reached a maximum of 41°/s. Accelerometers were mounted on the 3D-printed platform to measure the acceleration of the PK-Eye™ models when in motion. The accelerometers 622B01 had a sensitivity of 100 mV/g with a range of ± 490 m^2^/s. Temperature sensors PT-100 were also mounted on the platform around the models to measure the temperature across a wide range of locations. PT-100 measurements ranged from −50°C to 250 °C with a tolerance of ±0.15 + 0.002 × temp (in °C). The program variables (acceleration and temperature) and graph readouts were recorded (drug release with and without eye movements have been previously reported [25,26]).

##### Automating Drug Concentration Readout with a Concentration Probe Setup

An Easy Switch Solutions™ Fluid Handling platform (ESS™) from Fluigent (Le Kremlin-Bicêtre) [35] was included in the system to achieve real-time monitoring of drug release profiles between either multiple models or fluid inlets. The ESS™ platform had the ability to specifically select the outflow from different models/inlets into the concentration detector to obtain different release profile readouts. The ESS™ included (a) three 3-port/2-way microfluidic valves (2-SWITCH, an inlet port C and two outlet ports A and B), (b) an 11-port/10-position rotary valve (M-SWITCH, inlet port positions 1–10 and an outlet port), and (c) a SWITCHBOARD. The 2-SWITCH components and the M-SWITCH were powered and linked by the SWITCHBOARD.

1. Demonstrating setup capability of concentration probe

In a first experiment to demonstrate the capability of the setup, three inlet flow rates were set at 1.5, 2.0 and 2.5 µL/min flowing into three 2-SWITCH components that were connected to the M-SWITCH. The program was written to toggle the 2-SWITCH and M-SWITCH every 10 min to switch among three flows for 1 h. The rate of the M-SWITCH outflow was recorded via the flow unit connected to the outlet of the M-SWITCH. The flow rates of all flow units were recorded and analysed by MATLAB.

2. Quantification of a labelled protein with the concentration probe setup

A thermoelectrically cooled, high sensitivity spectrometer was installed and integrated with the microfluidic setup and was then used to measure the fluorescence of albumin-conjugated Alexa Fluor 488 (Alexa albumin). The spectrometer QEPRO-ABS was connected to the light source LSM-470A. Two QP600-1-UV-VIS optical fibres connected the flow cell where Alexa albumin was quantified to the light source and the spectrometer QEPRO-ABS (concentration probe). A calibration curve was obtained with Alexa albumin (3.9–62.5 μg/mL) and the integration window for the programme was fixed at 250 ms, with each concentration reading conducted at a rate of 1 measurement per second. A negative control was performed with PBS, pH 7.4 and post-processing of data was conducted using MATLAB, with the area under the curve (AUC) obtained for each signal converted to concentration with the extinction coefficient. The protein was then injected (5.0 mg/mL, 100 µL) into the posterior cavity of the PK-Eye™ model without membrane, connected directly to the concentration probe. With one measurement taken per minute through the probe, a concentration-time curve for the protein cleared from the model was obtained in real-time.

In a second experiment to demonstrate the integration of the ESS™ platform with the concentration probe, the 2-SWITCH was connected to one PK-Eye™ model with membrane, with outlet ports B and A connected to the M-SWITCH and waste respectively. PBS, pH 7.4 was pumped into port C of the 2-SWITCH through the model, and the outflow was switched between ports A and B. Port B of the 2-SWITCH was connected to position 1 of the M-SWITCH, while a PBS reservoir was connected to position 10 of the M-SWITCH. Alexa albumin was then injected (5.0 mg/mL, 100 µL) in the posterior cavity of the model. The program toggled the 2-SWITCH and the M-SWITCH every 2 h accordingly to select fluid from either the model or the PBS reservoir to be detected by the concentration probe in real-time. The AUC for each signal was converted to concentration (each signal was taken at a rate of 1 measurement per minute over 5 days) and a concentration-time profile was obtained by data processing with MATLAB.

### 2.3. Data Analysis

All results are presented as the mean and standard deviation (±STD) unless stated otherwise. Data were post-processed using MATLAB_R2022A, MathWorks. The program automatically read and assigned each data column to a variable and plotted them along pre-defined axes. Real-time monitoring of labelled protein was conducted using the software OceanView 2.0.8.

## 3. Results

### 3.1. Modelling Experiments with the Microfluidic System

#### 3.1.1. Choosing the Right Hyaloid Membrane Part to Separate the Anterior and Posterior Parts of the Posterior Flow Model

**Figure 3:**
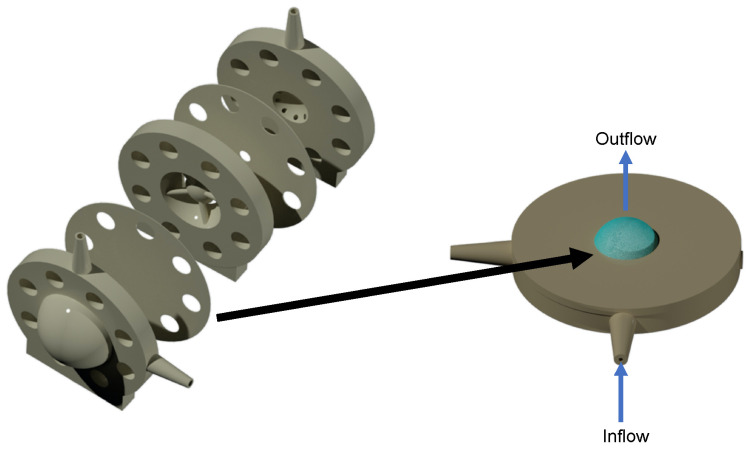
Modelling of the hyaloid membrane. (**left**) The posterior inflow model and (**right**) the pursing model. The hyaloid membrane was clamped between the two parts of the pursing model and was pursed under pressure with the fluid flowing through the membrane (from bottom to top). The pursing of the membrane (highlighted in blue) in the pursing model with blue arrows indicate the direction of the flow.

The hyaloid membrane (Figure 3) is made of a Visking material, which created a partition between the anterior and posterior cavities to separate the content of the posterior cavity from the anterior cavity [36]. The Visking membrane is a polymeric membrane and was used to filter drugs between the two cavities to create a flow resistance that pressurised the model within physiological parameters (between 10 and 20 mmHg) [37]. Polymer membranes have found numerous uses in a number of industries, including pharmaceutical industry applications for drug delivery and filtration [38,39,40]. Their diverse properties, including hydrophilicity/hydrophobicity, biocompatibility and permeability, make them uniquely suitable for these applications. This design concept of partitioning the two cavities was first demonstrated with the first generation PK-Eye™ model [22], which was eventually used to design the follow-up prototype, called the posterior inflow model [25].

Understanding their flow and transport characteristics is thus key for their use and is significantly dependent on the material structure [40]. The concept and importance of aqueous flow to study drug clearance in the PK-Eye™ model has previously been demonstrated for both proteins and small molecules from a pharmaceutical science perspective [22,23,24,25,26,27,28,41]. In this report, membrane permeability and its effect on pressure-flow are important in modelling, where cavity separation and molecule filtration based on size were further assessed from an engineering perspective. The posterior part of the model was modelled into a simple two-part pursing model to study the pressure and flow of different hyaloid membrane sizes and cut-offs. The permeability of different cellulose membranes for applications in the PK-Eye™ model was also assessed with these pursing models. The pressure drop through membrane filtration is given by the Weissberg-Sampson-Poiseuille approximation (Equation (1)) factoring in resistance created by a cellulose polymer membrane, which varies depending on pore size (MWCO) and the surface area of contact. As molecules used ocularly vary in molecular size (from small molecules of a few Da to antibodies up to 150 kDa), membranes with molecular weight cut-offs of 12–14 kDa, 50 kDa and 300 kDa were specifically assessed in this study, with surface areas of 5.0 to 20.0 mm, and the relationship between pressure and flow rate was determined in each case.

(1) $$\Delta p=\frac{8µTQ}{{\pi R}^{4}}+\frac{3µQ}{R^{3}}$$

where Δ*p* is the pressure drop, *µ* is the shear viscosity of the fluid, *T* is the thickness of the membrane, *Q* is the flow rate and *R* is the radius of the pore.

#### 3.1.2. Selecting the Right Membrane Diameter Size

**Figure 4:**
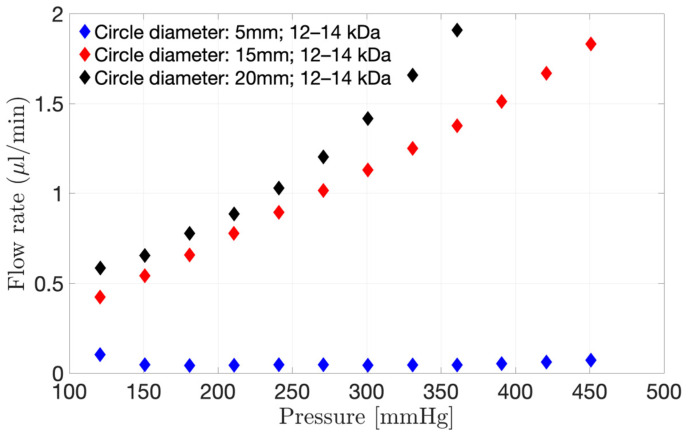
Interstitial flow rate through the 12–14 kDa membrane when using the pursing model for a range of pressure varying from 0 to 451 mmHg. Three different surface areas were investigated, i.e., 5.0, 15.0 and 20.0 mm diameter circles.

Three different diameters of a 12–14 kDa membrane were clamped on the pursing model (Figure 4). An increase in exposed surface area (Figure 4) from 5.0 to 20.0 mm diameter resulted in an increase in flow through the model at a fixed pressure. For example, at 150 mmHg, the flow rate through the membrane was 0.66 µL/min for a circular area of 20.0 mm diameter, decreasing to 0.54 µL/min for a diameter of 15.0 mm and to 0.05 µL/min for a diameter of 5.0 mm. Therefore, it is very important to get the dimensions of the model as close as possible to the real eye when modelling the hyaloid membrane of the PK-Eye™ in order to help assess the flow through the membrane prior to conducting an in vitro study.

#### 3.1.3. Selecting the Right Membrane Pore Size

**Figure 5:**
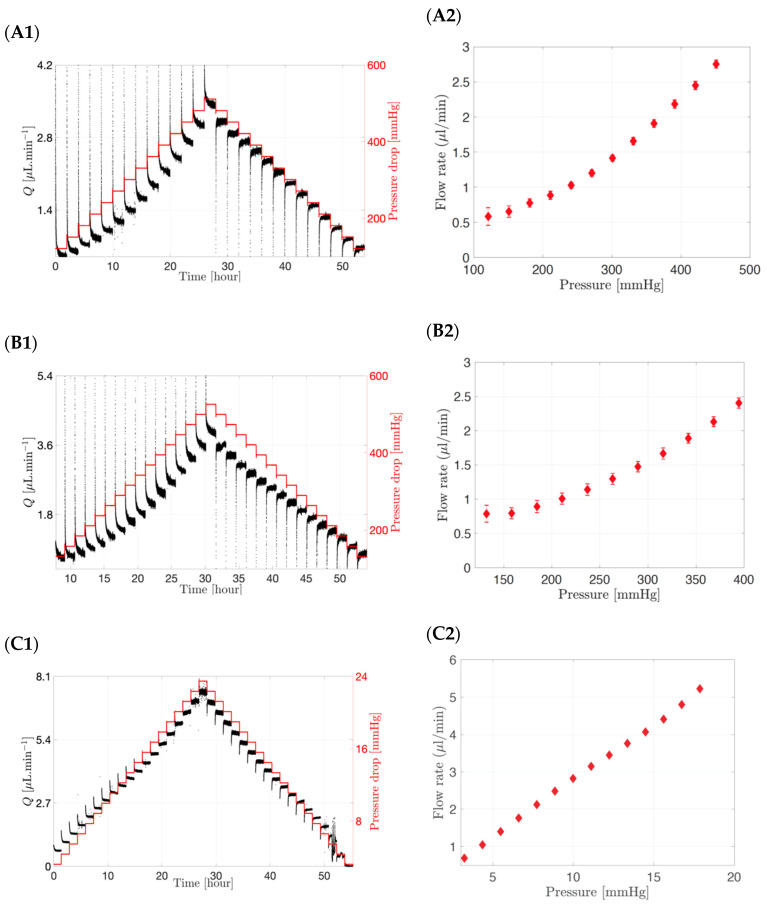
(**A1**,**A2**) Interstitial flow rate and pressure drop through a 20.0 mm diameter circular surface for the following pore size membrane: (**A1**,**A2**) 12–14 kDa, (**B1**,**B2**) 50 kDa and (**C1**,**C2**) 300 kDa. Flow rate increases with increasing pressure and membrane MWCO.

The interstitial flow rates through different membranes (12–14 kDa to 300 kDa) and a fixed surface area (20.0 mm diameter) using a range of pressures are shown in Figure 5. In the left panel, both the flow rate and pressure drop were plotted over a period of 50 to 60 h, while in the right panel the flow rate versus the pressure obtained from the left panel are shown with their standard deviations. The pressure was first increased in a stepwise manner and then decreased in a stepwise manner to minimise any hysteresis effects. The graphs (Figure 5A1,B1,C1) showed a direct incremental relationship between the pressure drop (in red) and the flow rate (in black) in the model, irrespective of membrane pore size.

Lowering the MWCO of the membrane resulted in a higher pressure drop. For example, a flow rate of approximately 2.0 µL/min was obtained at a pressure drop of 361 mmHg for a 12–14 kDa membrane (Figure 5A2), decreasing to 342 mmHg for a membrane with a MWCO of 50 kDa (Figure 5B2) and to approximately 7 mmHg for a membrane with a MWCO of 300 kDa (Figure 5C2) for a fixed surface area corresponding to a circular section of 20.0 mm diameter. When a 50 µL injection of bevacizumab (25.0 mg/mL concentration) was injected in the SVF of the posterior inflow model with a 300 kDa membrane as the hyaloid membrane, it resulted in a pressure of less than 10 mmHg in the posterior chamber. Therefore, it can be concluded that, for membranes with a small pore size (12–14 kDa to 50 kDa), non-physiological pressure values of up to 361 mmHg are required to reach the physiologically relevant flow rate (2.0 µL/min). An increase in MWCO and thus pore size, however, resulted in an increase in permeability and flux through the membrane, implying that lower pressure values will be required to produce a flow rate of 2.0 µL/min (7 mmHg for 300 kDa membrane). In addition, small surface areas irrespective of pore size would result in the higher pressures needed to achieve 2.0 µL/min flow rates.

The pursing studies demonstrated the relationship between a pressurised hyaloid membrane and interstitial flow rate by simplifying the PK-Eye™ model into a two-part pursing model clamping hyaloid membranes of different pore sizes to different outlet circumferences. The interstitial flow rate was reported for a range of pressures demonstrating the importance of a pressurised model. These studies also emphasised the importance of controlling pressure in relation to the elasticity of the hyaloid membrane. Experimental evidence indicated that the assessed membranes were able to maintain their elasticity within specific pressure ranges, beyond which the membranes would “break” due to high pressure. Thus, the choice of membrane and exposed surface area to flow are critical parameters in model design, while the careful choice of pressure in a microfluidic system for modelling is essential to the assurance of membrane integrity throughout the testing period.

### 3.2. General Platform Setup

Table 1 demonstrates the evolution of the PK-Eye™ prototypes with the creation of a real-time monitoring platform associated with the newer prototypes. The first-generation PK-Eye™ model was the first in vitro ocular model that introduced two main cavities (anterior and posterior cavities) separated by a Visking membrane (to separate the contents in the two cavities). The model had an inlet port located at the posterior part of the membrane, and an anterior outlet port to sample the drug release [22]. This model helped evaluate drug clearance of various intravitreally injected small and large molecules [22,23,24,27,28], with the use of a peristaltic pump, and by sampling drug release from the outflow port and analysing it with high performance liquid chromatography (HPLC) or another quantification assay. Initial studies with the first-generation PK-Eye™ and with the peristaltic pump setup (flow rate of 2.0 µL/min) demonstrated in vitro protein clearance at their clinical doses to be similar to half-life values reported in vivo (i.e., in human eyes): for example, a dose of 0.5 mg of ranibizumab and 1.25 mg of bevacizumab demonstrated in vitro half-life values of 8.1 ± 3.1 days [22] and 10.1 ± 0.7 days [28], respectively; where in vivo values (human eyes) were 7.2 [42]–9.0 [43] days and 6.7–10.0 days [44,45,46,47], respectively. However, some limitations of the model setup with the peristaltic pump included lack of scale-up capabilities and difficulty in determining batch-to-batch reproducibility. This simplified model setup was adequate to broadly understand drug kinetics under the effect of posterior flow but did not have the capabilities to monitor pressure-flow, eye movements and real-time clearance in an advanced manner, which are all essential in ocular drug testing. In this manuscript, the first-generation PK-Eye™ model was only used in the initial troubleshooting and validation phase to understand and further develop the real-time monitoring platform.

The first-generation model was then further developed into a posterior inflow model and a ciliary inflow model, where the flow and port alignment have been previously reported [25]. Both these prototypes contained the same two cavities, but with a more intricate anterior cavity, i.e., addition of an iris part, a lens part, and a hyaloid membrane, with the inlet port located before the lens layer [25]. Similar to the first-generation model, the elimination pathway designed with these models was the anterior hyaloid pathway, which has been reported as the predominant pathway for biologics and solubilised small molecules [22]. Both these models have previously been used to study drug clearance of both small and large molecules [25]. These models were used in this manuscript to conduct proof-of-concept experiments to demonstrate the real-time monitoring capabilities of the platform.

Other more advanced models have been developed to further assess the capabilities of the real-time monitoring platform. The design of both the posterior inflow model and the ciliary inflow model was used to build an additional model called the RCS model (Table 1) [25]. The model has similar cavities to the ciliary inflow model (inlet port after the lens part of the model) but has a second set of inlet/outlet ports at the back of the model. The RCS model was the first designed to study the drug clearance of a small or large molecule eliminating simultaneously from the front and the back outflow ports in real-time. The inlet flow at the back of the model allowed the transfer of drug from the RCS membrane to the collecting reservoir connected to the posterior outlet port [25]. As compared to the first-generation model, the RCS model previously displayed in vitro drug clearance of small molecules that were relatively closer to in vivo values, demonstrating an advancement in the design of the model closely related to a real human eye [25]. In addition, the effect of eye movements from two elimination pathways was also first demonstrated with the RCS model. Here, this model was used to understand the advanced capabilities of the microfluidics in testing the pressure of two membranes [25], which have not been previously demonstrated. Though not shown in this current manuscript, the RCS model helped develop the intracameral model (Table 1) [26], which has been used to extensively understand the capabilities of the microfluidic system and the eye movement platform for intracamerally administered small molecules.

### 3.3. Automating Flow with Microfluidics

#### 3.3.1. Scale up Capabilities of the Microfluidic System

Traditional syringe pumps or peristaltic pumps are commonly used in pharmaceutical labs and work for imposing a constant flow rate through a setup. The mechanical movement generating the flow rate usually results in low cyclical flow rate variations throughout the course of the test. A pressure-controlled flow system, as opposed to syringe pumps or peristaltic pumps, was introduced to allow large scale experiments by allowing a single pressure source to control several models simultaneously. The flow rate controlled by compressed air results in an extremely smooth flow rate with quasi-null flow rate variation. Moreover, the response of the system to any change in pressure or flow rate is smooth and fast, allowing for a more dynamic system. Another advantage is that, while troubleshooting one faulty pump in a microfluidic system, it is easier to isolate the pump from the system and rectify the issue than with the peristaltic pump. Moreover, the pulsatile flow provided by peristaltic pumps often fluctuates due to loss of interaction between the rollers and tubes. Microfluidic pumps, however, have been designed to reduce flow rate variability to allow greater control of flow through pressure-driven pumps [48].

**Figure 6:**
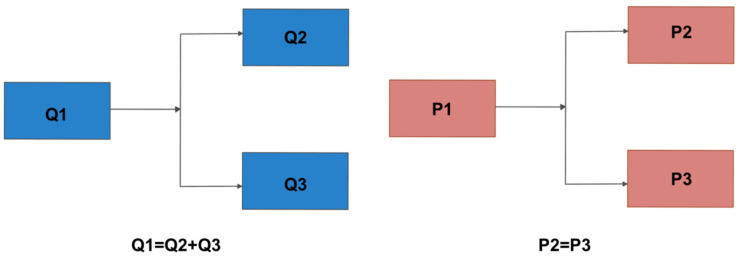
Schematic representation of setup mounted in parallel using (**left panel**) flow rate (Q) and (**right panel**) pressure (P) controlled system. The microfluidic setup reported in the manuscript incorporated a pressure-controlled system.

The microfluidic system can broadly be sub-divided into pressure controller (using a microfluidic controller called FlowEZ) and flow rate sensors (called flow unit S). The system was controlled by a computer that allowed either flow rate control or pressure-based control. Flow rate divides in systems in parallel and, therefore, only one model can be controlled with a set flow rate (left panel, Figure 6). On the other hand, pressure does not change across a parallel system and several models can be controlled simultaneously if mounted in parallel from a single pressure source. This is a large advantage in a pressure-controlled system, since it can feed different models from a single pressure source when mounted in parallel. The flow rate divides into each branch of the system, while the pressure stays the same when a system is mounted in parallel (right panel, Figure 6). Therefore, it is possible to scale up a fluidic system using a single pressure source (pump) to feed different models mounted in parallel.

**Figure 7:**
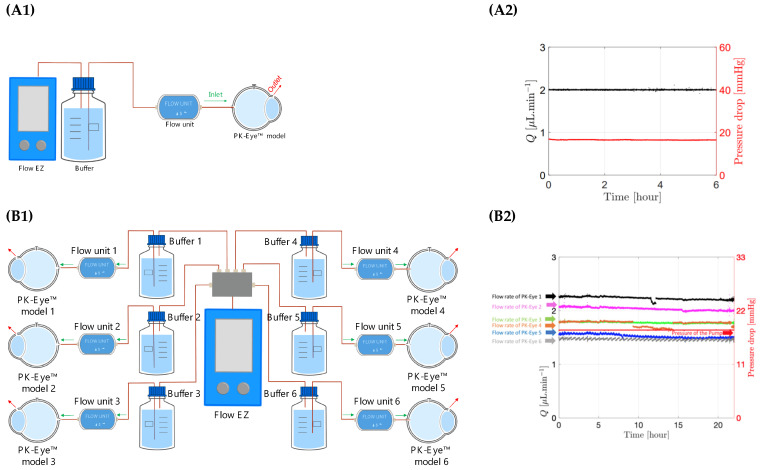
(**A1**,**A2**) Schematic representation of flow rate control using first generation PK-Eye™ models in PBS at room temperature (RT, ~20–22°C) with a pressure control of approximately 20 mmHg for 6 h. (**B1**,**B2**) Schematic representation of pressure control with a 1:6 ratios using first generation PK-Eye™ models in PBS at RT (~20–22°C) with a pressure control of 18 mmHg for 24 h.

A flow rate-controlled system was used by connecting one model to one pressure controller (Figure 7A1,A2). The flow rate was set at 2.0 μL/min with the pressure automatically adjusted by the system and the model successfully ran at the corresponding flow rate throughout the testing period at a data recording frequency of 1 Hz. The maximum number of pressure controllers that could run together on one system corresponded to 6 × pressure controllers coupled with 6 × flow rate sensors and each flow rate sensor connected to one PK-Eye™ model. The maximum data recording frequency of the system is 50 Hz, which corresponds to a maximum of 1800 data points per second (50 points per second × 6 pumps × 6 flow rates).

Ratio experiments (Figure 7B1,B2) were then conducted to demonstrate scalability of the new equipment by connecting one pressure controller (Flow EZ) to a maximum of six models mounted in parallel. The 1 to 6 ratio experiment consisted of 1 × pressure controller to 6 × different flow rate sensor units each connected to 1 PK-Eye™ model per flow unit sensor. The pressure controller was set at a constant value and the flow rates through each PK-Eye™ were recorded (Figure 7B2). Each model flowed at a slightly different flow rate but within the natural aqueous humour flow rate range, which usually varies between 1.5 μL/min at night and 3.0 μL/min in the morning [49]. The minimum and maximum flow rates were 1.5 μL/min and 2.7 μL/min, respectively. This demonstrates not only that a single pressure controller can control 6 × PK-Eye™ models, but that the PK-Eye™ model, tubing and flow connector setups are relatively identical to each other. Any hardware variations that could result in a few μL/min difference between each setup were not observed.

#### 3.3.2. Circadian Rhythm Using the Microfluidic System

**Figure 8:**
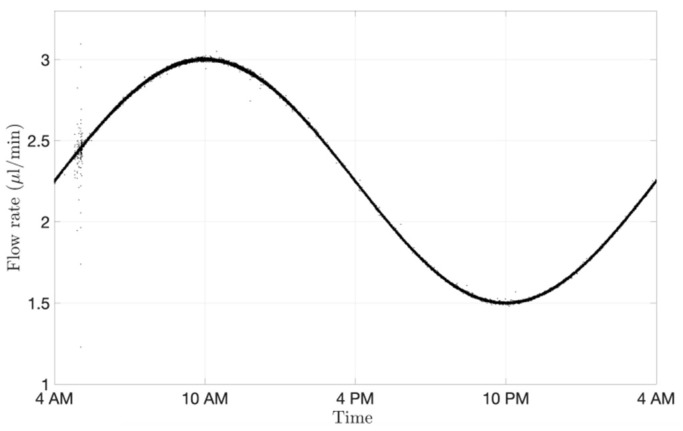
Circadian rhythm graph readout from the microfluidic system processed with MATLAB and shown over a period of 24 h. The microfluidic system reproduced the physiological aqueous humour flow rate fluctuations.

The microfluidic setup cannot only impose a constant flow rate, but it can also vary the flow rate in time to reproduce mass exchange more closely within the eye. Aqueous flow is secreted at 1.5–3.0 μL/min at the ciliary body level. The flow rate differs in the morning (3.0 μL/min), afternoon (2.4 μL/min) and night (1.5 μL/min) [49,50]. Circadian rhythm (Figure 8) was achieved with the microfluidics by generating a sinusoidal wave ranging from 1.5 to 3.0 µL/min within a period of 24 h. The signal was recorded with a frequency of 1 Hz. The change of flow rate during the day and night takes into account the fluctuations of flow convection in the eye, which could potentially contribute to changes in ocular drug clearance time.

#### 3.3.3. Demonstrating QC with Model Setup

**Figure 9:**
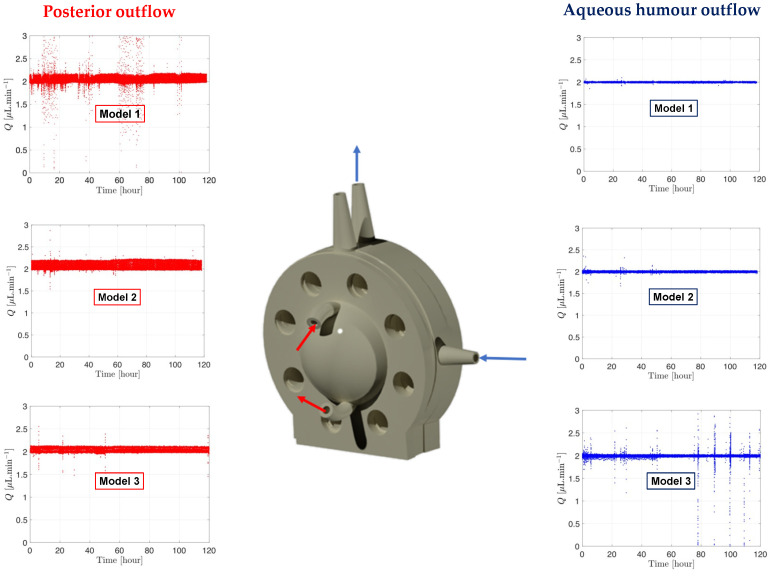
Graph readout from the microfluidic system processed by MATLAB showing the flow and pressure from the RCS models with SVF at 37°C. The direction of the aqueous humour and posterior outflows are shown on the RCS 3D rendering model. **Red** is associated with posterior fluid flow rate shown on the left panel and **blue** to aqueous humour flow rate shown on the right panel.

The microfluidic setup coupled with an assembled PK-Eye™ model can help demonstrate batch-to-batch reproducibility between experimental arms and can provide an indication of SVF leakage during the testing period. Preventing SVF leakage from the posterior cavity to any outflow ports or the anterior cavity will ensure QC and retain the SVF for the duration of in vitro ocular testing. A spike in pressure or blockage of the ports from SVF is a good indication of SVF leakage. Ciliary inflow models and RCS models were developed [25] that introduced more intricacy in model design (especially the RCS model). The RCS model was designed to have two simultaneous pathways to study drug elimination from the front (anterior hyaloid pathway) and from the back (RCS pathway), and introduced the use of two membranes of different MWCOs (i.e., 300 kDa vs. 12–14 kDa respectively). Stability of pressure-flow using two membranes and the use of SVF were investigated with the RCS models (Figure 9).

RCS models were assembled with a fresh batch of SVF, and pressure was monitored for 5 days (Figure 9). The aqueous humour outflow (right panel, Figure 9) is shown in blue with the blue arrows on the model indicating the direction of the flow. A second flow at 2.0 µL/min (left panel, Figure 9) was also established in the back of the model, shown in red, with red arrows indicating the flow direction through the model. The flow did not enter the posterior cavity and simply flowed along the posterior side of the RCS membrane akin to the RCS blood flow to collect the drug and carry it into the reservoir for drug characterisation. It can be noticed that the flow rate data remained constant at 2.0 µL/min and stable for the duration of the testing period, with only minor fluctuations in flow rate (slight deviations from the flow rate baseline of 2.0 µL/min). This indicated no issues with SVF leakage from the model and emphasised the general robustness of the microfluidic system and model setup across the entire duration of the experiment.

### 3.4. Automating Eye Movement with a Novel Eye Movement Platform

#### 3.4.1. Building the Eye Movement Platform

Temperature sensors and accelerometers were acquired and integrated with the in-house eye movement platform. These different components were supplied by different manufacturers and were integrated into a unique platform from NI to help with data collection and synchronisation. The platform acquired and generated multiple signals simultaneously with frequencies in the Megahertz range. The cRIO-9048 acted as a computer working on a Linux Real System enabling a high data transfer rate, which is multiple times higher than traditional computers. The cRIO-9048 accommodated eight different cards to acquire specific signals, i.e., for the accelerometers, the temperature probes, the rotary motor and a card to generate Transistor-Transistor Logic (TTL) signals for future integration. Preliminary work focused on understanding the compatibility of each card with each sensor as well as eventual hardware connections and programming.

**Figure 10:**
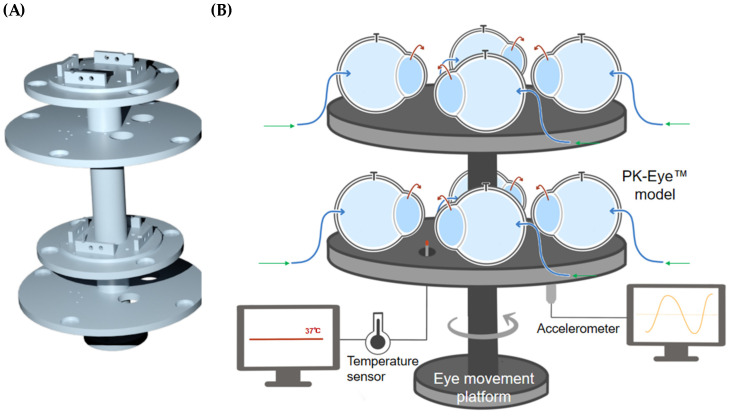
(**A**) 3D rendering of the 3D printed eye movement platform with a (**B**) schematic of the platform holding 8 × PK-Eye™ models connected through tubing to the microfluidic equipment.

The rotation stage V-611 had a high load capacity to hold the platform and the PK-Eye™ models (Figure 10A) and generated a high velocity and acceleration to mimic the chosen eye movement. The sensor had a very high resolution (0.0001°) in order to reproduce micro-saccades with a maximum velocity of 3000°/s and a maximum load of 10 kg-force. The platform was 3D printed using the engineering Tough 2000 resin from Formlabs to hold the models and the different sensors (temperatures and accelerometers). Each model was composed of multi-compartments that were screwed together to the platform. The models were mounted on top of the platform while the sensors were mounted below the top surfaces to avoid obstructing the positioning of the models (Figure 10B).

**Figure 11:**
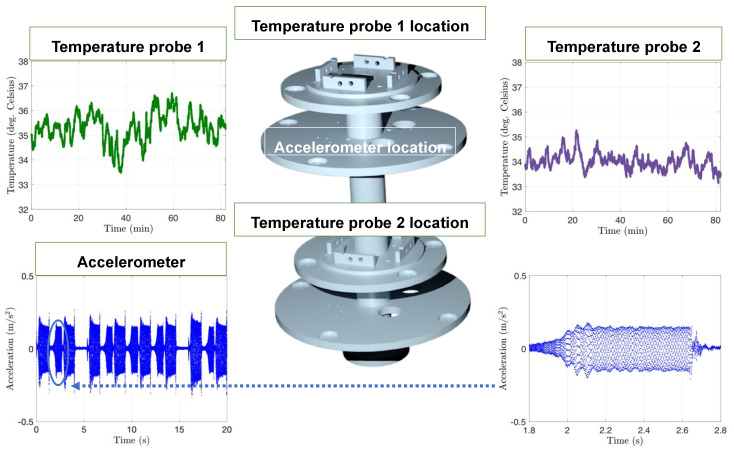
Graphs of the accelerometer and temperature readings with the platform 3D rendering showing the location of each probe. **Dark green** corresponds to the temperature probe 1, **purple** corresponds to the temperature probe 2 and **blue** corresponds to the accelerometer. The temperature and acceleration sensors were recorded simultaneously using Labview and NI. An example of acceleration is plotted over 20 s and over a shorter period of 1 s to show the 6400 points per second of recording. Temperature sensors were recorded at a lower frequency of 1 Hz. The placement of the different vivarium lamps around the models ensured a uniform temperature across the platform.

The accelerometers acquired are routinely used in industry when precise motion data are required with a sensitivity of 100 mV/g. The accelerometers were connected to the card 9230 from the NI-cRIO-9048, which had a maximum capacity of three accelerometers recorded simultaneously. The data monitored by the accelerometers when the platform was in movement using Labview and NI are shown in Figure 11. The reading of the signal was in real-time and corresponded to 6400 data points per second or a frequency of 6.4 kHz. The next type of sensor was the thermocouple that ensured that the models were at a constant temperature of 37.0 ± 2.0 °C. Card 9216 was acquired for the NI-cRIO-9048 to connect to the eight temperature sensors. Two temperature sensors PT-100 were arranged around the platform. As a heat source, vivarium lamps were used instead of a traditional heating plate to simplify the setup for the eye movement platform. Temperature across the two temperature sensors was within 1.5 °C, demonstrating uniform heating of the PK-Eye™ models. The two temperature sensors recorded temperatures of 35.3 ± 0.6 °C (probe 1) and 34.0 ± 0.3 °C (probe 2) (Figure 11).

#### 3.4.2. Programming the Eye Movements

**Figure 12:**
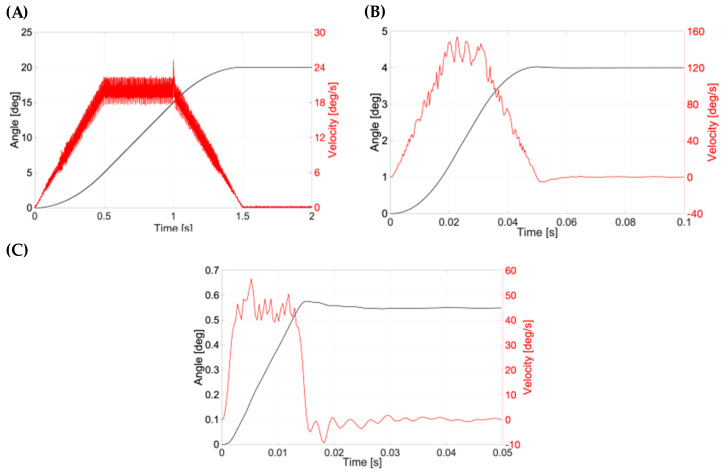
Typical graph for a (**A**) smooth pursuit, (**B**) saccadic and (**C**) micro-saccadic movement connected to the PK-Eye™ model.

Eye movement parameters were inputted (Table 2, Figure 12), and demonstrated using the models, temperature sensors, accelerometers and vivarium lamps, with the ciliary inflow model. The program reported for the eye movements, i.e., smooth/slow pursuit, saccades, and micro- saccades, is similar to the eye movements in the human eye [51,52,53,54,55]. The smooth/slow pursuit is a continuous movement of the eye without a break that attains a maximum speed of 87°/s [52] (range: 20–40°/s [56]). Saccadic eye movements involve faster rotation of the eye followed by a short period of rest: for example, silent reading involves 2°-saccades in 30 ms with a mean fixation duration period of 225 ms and scene perception attains 4–5° covered in 40–50 ms with a 330 ms fixation duration period [57]. Micro-saccadic eye movements correspond to visual fixation and have a low magnitude of the angle displacement (0.55 ± 0.07°) covered in a short time frame (13 ms) that is repeated at an average of 1.25 s [51]. The effect of these three eye movements has been previously demonstrated in vitro with intracameral small molecules (timolol and brimonidine) [26]. The release of intravitreally injected small molecules and biologics in the posterior inflow model, ciliary inflow model and RCS model has also been extensively demonstrated [25].

Here, the real position and velocity of the key motion were further assessed (Figure 12). The position in degrees and the velocity (°/s) for the smooth pursuit are shown in Figure 12A. The velocity of the PK-Eye™ model is shown in red, while the angle is shown in black. The data recording was done at 2000 Hz. The PK-Eye™ model covered 20°/1.5 s with a peak velocity at 25.18°/s. The smooth pursuit saccades movement is shown in Figure 12B (recorded at a rate of 2847 Hz), where a saccadic motion of 4°/50 ms with a peak velocity of 153.8°/s was computed. The micro-saccades movement is shown in Figure 12C, and had a displacement of 0.55°/14 ms with a peak velocity of 52.5°/s. These results, for the first time, demonstrate the programming of the eye movement in more detail (Table 2) to evaluate and assess the real-time monitoring of eye movements, which can further be used to evaluate in vitro properties of other drug molecules and formulations of interest.

### 3.5. Automating Drug Concentration Readout with a Concentration Probe Setup

#### 3.5.1. Demonstrating Setup Capability of Concentration Probe

**Figure 13:**
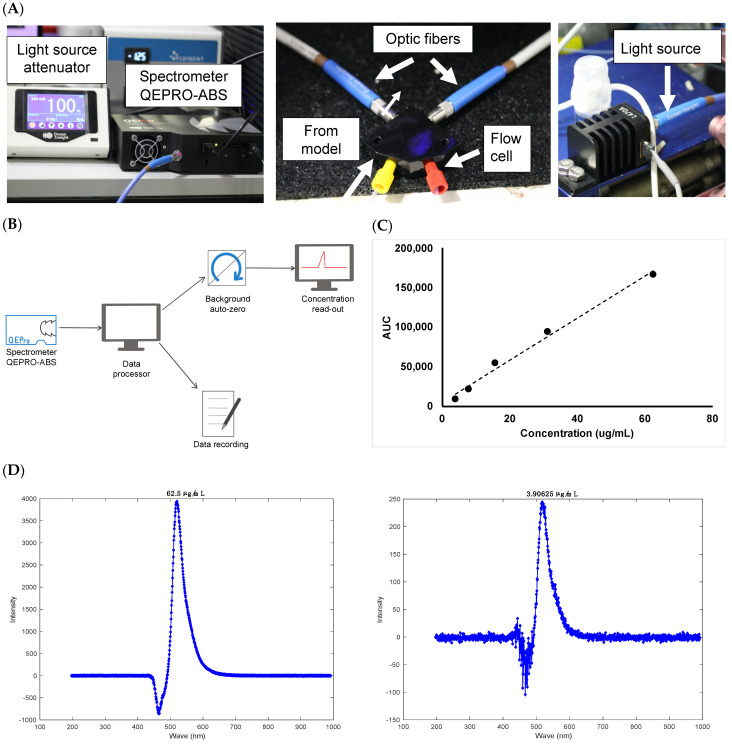
(**A**) Setup of the concentration probe to achieve real-time monitoring of concentration, (**B**) schematic of the concentration probe program, (**C**) calibration curve conducted with Alexa albumin (3.9–62.5 μg/mL) and (**D**) representative chromatograms of Alexa albumin at (left panel) 62.5 μg/mL and (right panel) 3.91 μg/mL.

There remains a need for analytical tools to accurately collect pharmacokinetic data in real-time to understand stability and drug release profiles. Real-time drug monitoring with the use of sensors and concentration probes would produce more accurate concentration-time profiles by reducing the time lag between sampling time points [58]. The use of UV fibre optic technology for in-situ dissolution testing has been a topic of interest in the last few years [59]. This offers precedent for the use of optic fibres for real-time assessment of drug clearance (akin to concentration determination in dissolution tests) in an in vitro ocular model. The concentration probe platform consisted of a spectrometer QEPRO-ABS with optical fibers QP600-1-UV-VIS acquired from Ocean Insight. The optical fibres were coupled with the ESS™ platform from Fluigent to allow continuous monitoring of several flow outlets with a single concentration probe (Figure 13). The continuous monitoring of this platform has been demonstrated for the first time and was operated autonomously using different programs from Ocean Insight and Fluigent, and did not require additional handling of the models.

The setup for the concentration probe is shown in Figure 13A with a schematic of the program used in Figure 13B. Combined without the need for manual sampling, the spectrometer demonstrated more data points, better stability and resolution at normal light levels as compared to other manual UV detection methods. Initial work focused on setting up the equipment and synchronising the system with the microfluidic setup. Significant troubleshooting was conducted to allow seamless real-time data collection and to avoid system crashing during the run. PBS, pH 7.4 was then injected into the flow cell of the concentration probe at 470 nm, which was taken as the “background” reading to zero the signal. Since extensive work on clearance studies in the model has been previously demonstrated (and compared to in vivo values) and referenced in this manuscript, Alexa albumin was chosen here as a molecule simply to demonstrate proof-of-concept of the concentration monitoring platform due to its higher extinction coefficient compared to unlabelled proteins. It was difficult to assess the concentration of unlabelled protein (e.g., ranibizumab and bevacizumab) with the concentration probe due to the sensitivity limit of the setup. As a result, Alexa albumin was chosen to demonstrate this novel setup with the in vitro model and was injected into the flow cell of the concentration probe at 470 nm. The AUC was calculated from the labelled protein signal obtained for each analysed concentration. The calibration curve generated good linearity with an R^2^ value of 0.9942 (Figure 13C). The plot was prepared using a stock solution of Alexa albumin (250 μg/mL) in PBS, pH 7.4 followed by serial dilutions (3.9–62.5 μg/mL, Figure 13D) that were quantified by the setup at 470 nm with a direct injection into the flow cell.

#### 3.5.2. Quantification of a Labelled Protein with the Concentration Probe Setup

**Figure 14:**
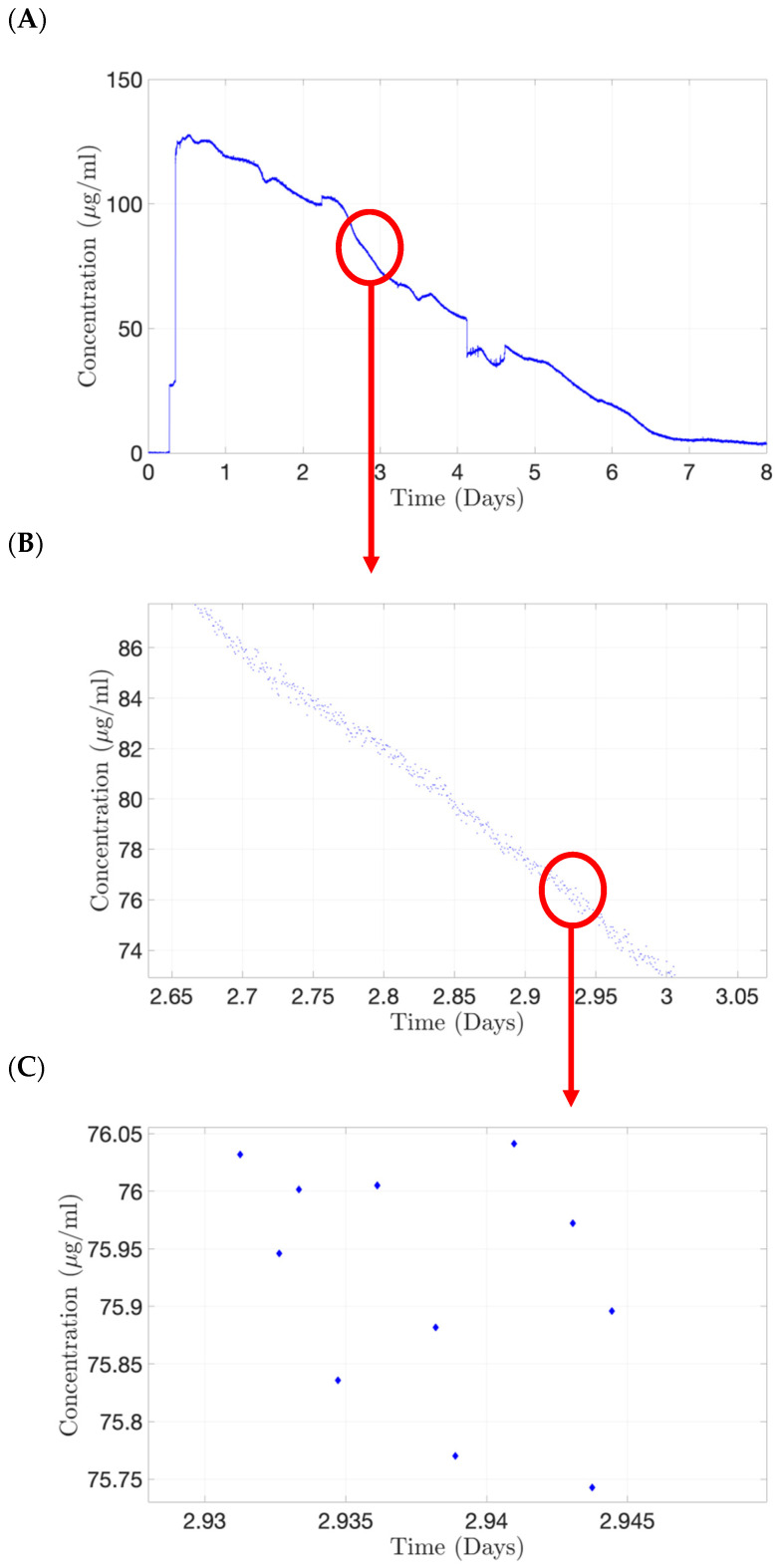
Continuous monitoring from the concentration probe using Alexa albumin (5.0 mg/mL, 100 µL) injected in posterior cavity of the first generation of the PK-Eye™ model. (**A**) Typical drug clearance readout, (**B**) zoomed version indicating stable readout from the concentration probe and (**C**) the variation of drug concentration detected by the probe is less than 0.3 µg/mL between each time point.

A preliminary experiment was conducted using a first-generation PK-Eye™ model, which was assembled without a membrane, with both cavities filled with PBS, pH 7.4. Alexa albumin (5.0 mg/mL, 100 µL) was injected in the posterior cavity of the model and the outlet port was connected to the flow cell of the concentration probe for real-time readout and allowed to run for 8 days (where protein concentrations were quite negligible) at a rate of 1 reading/minute (Figure 14). A typical drug release profile of the labelled protein (Alexa albumin) is shown in Figure 14A. A close-up of this profile from 2.65 to 3 days (Figure 14B) demonstrated the smooth decrease in drug concentration, indicating that the concentration probe was able to maintain a stable readout of the fluid passing through the flow cell. A further close-up from 2.93 to 2.95 days (Figure 14C) showed that the variation of drug concentration detected by the probe is less than 0.3 µg/mL between each time point.

**Figure 15:**
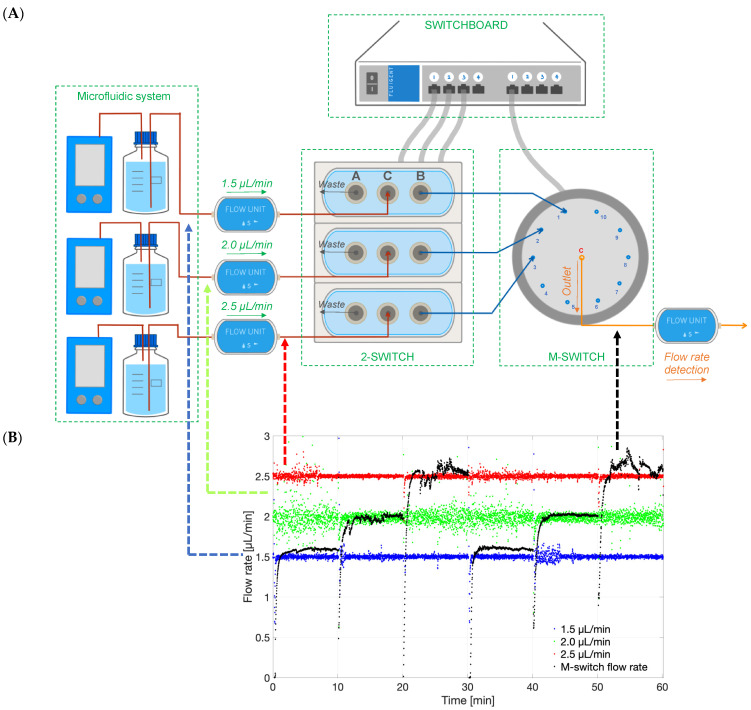
(**A**) Schematic of the ESS™ platform that could select different flow lines to be potentially analysed by concentration detection unit to monitor multiple PK-Eye™ models. The setup included a 2-SWITCH, a M-SWITCH and a SWITCHBOARD. Three flow units with pre-set flow rates (1.5, 2.0 and 2.5 µL/min) were connected to three 2-SWITCH, and the port B of the 2-SWITCH was connected to position 1, 2, and 3 of the M-SWITCH. A fourth flow unit (M-SWITCH flow rate) was connected to the outlet port C to record the flow rate selection between different outflows. (**B**) Graph of the different flow rates in time (1.5, 2.0 and 2.5 µL/min), and the M-switch unit flow rate (M-switch flow rate) demonstrating the selection of the flow rate through the M-switch.

The 2-SWITCH/M-SWITCH setup was integrated to ensure continuous drug quantification between multiple models after clearance from the PK-Eye™ model by using a connected concentration probe through the M-SWITCH (Figure 15A). This concept has been demonstrated for the first time in this manuscript to show the advancement of such a setup. The ESS™ platform successfully selected different fluid outlets flowing out from a combination of M-SWITCH and 2-SWITCH automatically (Figure 15B). Three different flow rates of 1.5, 2.0 and 2.5 µL/min were set to visualise the three different fluid outlets. The rate of the M-SWITCH outflow has been shown to automatically align with the inflow from the 3 × 2-SWITCH every 10 min as written in the toggle programme. Therefore, it provided the possibility to expand the accommodation capacity of the single concentration probe connected to realise the real-time monitoring of drug release between multiple flow outlets.

**Figure 16:**
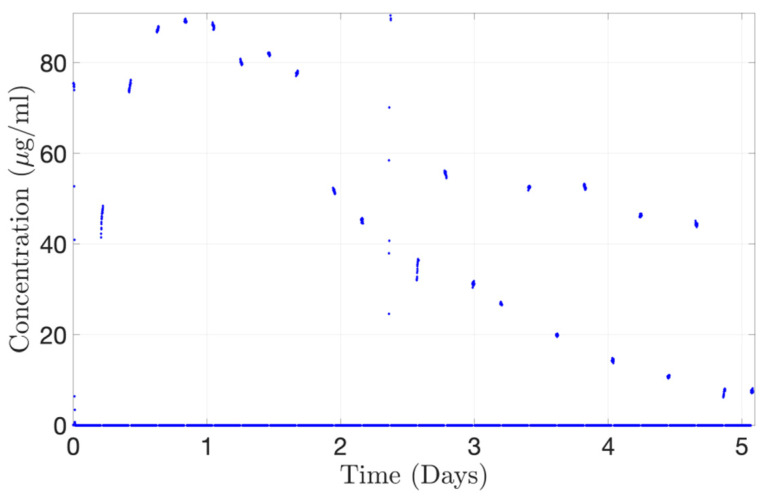
Continuous monitoring of Alexa albumin (5.0 mg/mL, 100 µL) from the concentration probe connected to the ESS™ platform toggling between the outlets from the posterior inflow PK-Eye™ model.

The concentration-time profile for Alexa albumin injected into the PK-Eye™ (posterior inflow model) was also determined with the concentration probe setup. The posterior inflow model was selected by the ESS™ platform to automatically run through the concentration probe for 20 min every 4 h 40 min. When not selected, the content from the posterior inflow model cleared into the waste reservoir, and PBS, pH 7.4 flowed through the concentration probe to avoid drug accumulation during the experiment. A toggling program was selected between the PK-Eye™ model and the PBS reservoir. The concentration probe was able to reproduce the drug release curve with a maximum concentration of approximately 89.0 µg/mL cleared from the model at 20 h (Figure 16). The gaps between points correspond to the 280-min interval when the drug was clearing into waste and not through the concentration probe, as a result of the toggling program. However, some points were out of range showing that, when handling concurrent multiple outlets through a single concentration probe, great care needs to be taken to ensure the probe is able to detect the right amount of drug. This demonstrates the automatic selection of channels flowing through the concentration probe, i.e., the posterior inflow model and a PBS channel. The program automatically selected the posterior inflow model and data were plotted (Figure 16). When not selected, the model did not stop flowing and the flow was automatically redirected to a waste reservoir. This set of data shows the potential for automatically redirecting different flows from the models via a concentration probe setup to remove any manual handling in order to record the concentration of cleared drug, and further illustrates the real-time monitoring capabilities of the platform.

The current concentration probe setup helped demonstrate proof-of-concept of a labelled protein, such as Alexa albumin. However, this also highlighted the main limitation of such setup, i.e., concentration quantification is only limited to labelled proteins. The majority of the drugs tested during preclinical development that are used in clinic are unlabelled proteins or small molecules. A labelled protein was chosen over an unlabelled molecule due to its high extinction coefficient and sensitivity ranges with the optic fibres. Ocular drugs, especially longer acting formulations, are usually injected at low doses and are meant to last for a few weeks/months (by releasing low drug concentrations). It was difficult to quantify such low concentrations with the current setup without protein labelling. Future work can focus on investigating different concentration probe systems that would be able to detect low concentrations of unlabelled drugs.

## 4. Conclusions

The PK-Eye™ is a pharmaceutical in vitro model of the human eye that can be used to fast-track the development of intraocular medicines. Different compartmentalised prototypes of the models were tested with a real-time automated platform to study various parameters that are relevant in pharmaceutical testing, such as drug distribution. The automated platform has the capability to record pressure drop, flow, temperature, eye movement and concentration in connected PK-Eye™ models in real-time. The microfluidic setup allows constant monitoring and control of aqueous flow, which can be used as a QC parameter to ensure that flow is not outside any specifications during the testing period. It can also be used as a monitoring tool to ensure that models are constantly pressurised throughout testing, as a leak in a model would result in a pressure value in the model close to zero mmHg, while the flow rate may remain unaltered. Fluid dynamics were also controlled to better replicate in vivo conditions e.g., the aqueous humour diurnal cycle, which could potentially affect drug biodistribution and release kinetics. One key parameter involved setting up computer-controlled microfluidics to mimic ocular aqueous outflow and achieve synchronisation between the models running in parallel with electronic data recorded simultaneously on single files at a rate of 1 Hz. Proof-of-concept experiments involved optimisation and troubleshooting of the flow rate within the models.

The eye movement platform was developed to study the different types of eye movements and their implications in drug clearance from the model. The design and development of the platform included the incorporation of temperature sensors and accelerometers for monitoring temperature changes and controlling movement, respectively. The platform has also been previously utilised to study the effect of different eye movements on the clearance of small and large molecules from the PK-Eye™. In addition, the integration of real-time concentration monitoring into the setup was an essential part of system automation, to reduce human intervention and the need for continuous sampling from models in in vitro clearance studies. The concentration probe was assembled and troubleshooted to achieve constant evaluation of drug clearance without the use of other routinely applied quantification methods, e.g., HPLC or micro-bicinchonic acid assay (microBCA). In addition, compared to manual sampling, multiple data points were generated per minute with the concentration probe setup in real-time. Proof-of-concept experiments were conducted with labelled proteins due to their higher extinction coefficients than those of unlabelled proteins, which allowed for higher sensitivity and detection of lower protein concentrations. The ESS™ platform was incorporated to further expand the accommodation capacity of the concentration monitoring system in order to provide the possibility of selecting different outlet flows and real-time monitoring of multiple models at the same time.

## 5. Patents

Improvements and design changes in the models are described in a patent specification [60], which lists S.A., Y.B., N.I., S.B. and P.T.K. are co-inventors.

## Figures and Tables

**Table 1:** Evolution of the PK-Eye™ model with a real-time monitoring platform.

Model-Platform	Models				
	First-Generation PK-Eye™ [22]	Posterior Inflow Model [25]	Ciliary Inflow Model [25]	RCS Model [25]	Intracameral Model [26]
*Model parts*					
Anterior cavity	Simple anterior cavity	Anterior cavity with iris part, lens part and hyaloid membrane			
Posterior cavity	Simple posterior cavity with SVF				
Membrane	One Visking membrane in the front			Two Visking membranes (one front and one back)	
Inflow	Posterior		Anterior		
Outflow	Front only (anterior hyaloid)			Front and back (anterior hyaloid and RCS)	
Anterior sampling in real-time	Yes				
Posterior sampling in real-time	No			Yes	
Molecules tested	Small and large molecules				Small molecules
*Platform*					
Flow	Peristaltic pump	Microfluidics			
Pressure-flow monitoring	No	Yes (with graph readout)			
Simultaneous models testing (*n*)	8	48			
Circadian rhythm	No	Yes (with graph readout)			
SVF leakage monitoring	No	Yes (with graph readout)			
Eye movement monitoring	No	Yes, one eye movement shown (with graph readout)			Yes, three eye movements shown (with graph readout)
Temperature monitoring	Thermometer	Temperature sensors			
Concentration readout	Manual sampling and HPLC analysis	Manual sampling and HPLC analysis, and use of concentration probe setup			

Abbreviations: HPLC: high performance liquid chromatography, RCS: retina-choroid-sclera and SVF: simulated vitreal fluid.

**Table 2:** Parameters for 3 movements programmed with the eye movement platform.

**Smooth pursuit**						
Movement 1	Movement 2	Movement 3	Movement 4	Movement 5	Movement 6	Movement 7
+20°/1.5 s	1 s pause	+20°/1.5 s	1 s pause	−20°/1.5 s	1 s pause	20°/1.5 s
**Scene pursuit saccades**						
Movement 1	Movement 2	Movement 3	Movement 4	Movement 5	Movement 6	Movement 7
+4°/50 ms	330 ms pause	+4°/50 ms	330 ms pause	+4°/50 ms	330 ms pause	−12°/1 s
**Micro saccades**						
Movement 1	Movement 2	Movement 3	Movement 4	Movement 5	Movement 6	Movement 7
+0.55°/14 ms	1.25 s pause	−0.55°/14 ms	1.25 s pause	+10°/500 ms	1.25 s pause	+0.55°/14 ms
Movement 8	Movement 9	Movement 10	Movement 11	Movement 12	Movement 13	Movement 14
1.25 s pause	−0.55°/14 ms	1.25 s pause	−20°/500 ms	−0.55°/14 ms	1.25 s pause	−0.55°/14 ms
Movement 15	Movement 16	Movement 17				
1.25 s pause	+10°/500 ms	1.25 s pause				

## Data Availability

Not applicable.

## References

[1] Ranta V., Urtti A. (2006). Transscleral drug delivery to the posterior eye: Prospects of pharmacokinetic modeling. Adv. Drug Deliv. Rev..

[2] Jager R.D., Aiello L.P., Patel S.C., Cunningham E.T. (2004). Risks of intravitreous injection: A comprehensive review. Retina.

[3] Thrimawithana T., Young S., Bunt C. (2011). Drug delivery to the posterior segment of the eye. Drug Discov. Today.

[4] Laude A., Tan L.E., Wilson C.G., Lascaratos G., Elashry M., Aslam T., Patton N., Dhillon B. (2010). Intravitreal therapy for neovascular age-related macular degeneration and inter-individual variations in vitreous pharmacokinetics. Prog. Retin. Eye Res..

[5] Awwad S., Henein C., Ibeanu N., Khaw P.T., Brocchini S. (2020). Preclinical challenges for developing long acting intravitreal medicines. Eur. J. Pharm. Biopharm..

[6] Kuentz M. (2015). Analytical technologies for real-time drug dissolution and precipitation testing on a small scale. J. Pharm. Pharmacol..

[7] Adrianto M.F., Annuryanti F., Wilson C.G., Sheshala R., Thakur R.R.S. (2021). In vitro dissolution testing models of ocular implants for posterior segment drug delivery. Drug Deliv. Transl. Res..

[8] Jones H.M., Gardner I.B., Collard W.T., Stanley P.J., Oxley P., Hosea N.A., Plowchalk D., Gernhardt S., Lin J., Dickins M. (2011). Simulation of human intravenous and oral pharmacokinetics of 21 diverse compounds using physiologically based pharmacokinetic modelling. Clin. Pharmacokinet..

[9] Lavé T., Chapman K., Goldsmith P., Rowland M. (2009). Human clearance prediction: Shifting the paradigm. Expert Opin. Drug Metab. Toxicol..

[10] Rostami-Hodjegan A., Tucker G.T. (2007). Simulation and prediction of in vivo drug metabolism in human populations from in vitro data. Nat. Rev. Drug Discov..

[11] Longest P.W., Tian G., Walenga R.L., Hindle M. (2012). Comparing MDI and DPI aerosol deposition using in vitro experiments and a new stochastic individual path (SIP) model of the conducting airways. Pharm. Res..

[12] Sun L., Cun D., Yuan B., Cui H., Xi H., Mu L., Chen Y., Liu C., Wang Z., Fang L. (2012). Formulation and in vitro/in vivo correlation of a drug-in-adhesive transdermal patch containing azasetron. J. Pharm. Sci..

[13] OhtoriI A., ToJo K. (1994). In vivo/in Vitro Correlation of Intravitreal Delivery of Drugs with the Help of Computer Simulation. Biol. Pharm. Bull..

[14] Barat A., Ruskin H.J., Crane M. (2006). Probabilistic methods for drug dissolution. Part 2. Modelling a soluble binary drug delivery system dissolving in vitro. Simul. Model. Pract. Theory.

[15] Bonam M., Christopher D., Cipolla D., Donovan B., Goodwin D., Holmes S., Lyapustina S., Mitchell J., Nichols S., Pettersson G. (2008). Minimizing variability of cascade impaction measurements in inhalers and nebulizers. AAPS PharmSciTech.

[16] Agu R.U., Ugwoke M.I. (2011). In vitro and in vivo testing methods for respiratory drug delivery. Expert Opin. Drug Deliv..

[17] Emami J. (2006). In vitro-in vivo correlation: From theory to applications. J. Pharm. Pharm. Sci..

[18] Choi S.H., Lionberger R.A. (2016). Clinical, Pharmacokinetic, and In Vitro Studies to Support Bioequivalence of Ophthalmic Drug Products. AAPS J..

[19] Patel S., Müller G., Stracke J.O., Altenburger U., Mahler H.-C., Jere D. (2015). Evaluation of protein drug stability with vitreous humor in a novel ex-vivo intraocular model. Eur. J. Pharm. Biopharm..

[20] Loch C., Bogdahn M., Stein S., Nagel S., Guthoff R., Weitschies W., Seidlitz A. (2013). Simulation of drug distribution in the vitreous body after local drug application into intact vitreous body and in progress of posterior vitreous detachment. J. Pharm. Sci..

[21] Auel T., Großmann L., Schulig L., Weitschies W., Seidlitz A. (2021). The EyeFlowCell: Development of a 3D-Printed Dissolution Test Setup for Intravitreal Dosage Forms. Pharmaceutics.

[22] Awwad S., Lockwood A., Brocchini S., Khaw P.T. (2015). The PK-Eye: A Novel in Vitro Ocular Flow Model for Use in Preclinical Drug Development. J. Pharm. Sci..

[23] Awwad S., Day R.M., Khaw P.T., Brocchini S., Fadda H.M. (2017). Sustained release ophthalmic dexamethasone: In vitro in vivo correlations derived from the PK-Eye. Int. J. Pharm..

[24] Awwad S., Abubakre A., Angkawinitwong U., Khaw P.T., Brocchini S. (2019). In situ antibody-loaded hydrogel for intravitreal delivery. Eur. J. Pharm. Sci..

[25] Velentza-Almpani A., Ibeanu N., Liu T., Redhead C., Khaw P.T., Brocchini S., Awwad S., Bouremel Y. (2022). Effects of Flow Hydrodynamics and Eye Movements on Intraocular Drug Clearance. Pharmaceutics.

[26] Liu T., Ibeanu N., Brocchini S., Khaw P.T., Bouremel Y., Awwad S. (2022). Development of an in vitro model to estimate mass transfer from the anterior cavity. Front. Drug Deliv..

[27] Egbu R., Brocchini S., Khaw P.T., Awwad S. (2018). Antibody loaded collapsible hyaluronic acid hydrogels for intraocular delivery. Eur. J. Pharm. Biopharm..

[28] Awwad S., Al-Shohani A., Khaw P.T., Brocchini S. (2018). Comparative Study of in Situ Loaded Antibody and PEG-Fab NIPAAM Gels. Macromol. Biosci..

[29] Sapino S., Peira E., Chirio D., Chindamo G., Guglielmo S., Oliaro-Bosso S., Barbero R., Vercelli C., Re G., Brunella V. (2019). Thermosensitive Nanocomposite Hydrogels for Intravitreal Delivery of Cefuroxime. Nanomaterials.

[30] Thakur S.S., Shenoy S., Suk J.S., Hanes J.S., Rupenthal I.D. (2020). Validation of hyaluronic acid-agar-based hydrogels as vitreous humor mimetics for in vitro drug and particle migration evaluations. Eur. J. Pharm. Biopharm..

[31] Chi Z., Azhar I., Khan H., Yang L., Feng Y. (2019). Automatic Dissolution Testing with High-Temporal Resolution for Both Immediate-Release and Fixed-Combination Drug Tablets. Sci. Rep..

[32] Tang J., Kong B., Wu H., Xu M., Wang Y., Wang Y., Zhao D., Zheng G. (2013). Carbon nanodots featuring efficient FRET for real-time monitoring of drug delivery and two-photon imaging. Adv. Mater..

[33] Zheng X.T., Chen P., Li C.M. (2012). Anticancer efficacy and subcellular site of action investigated by real-time monitoring of cellular responses to localized drug delivery in single cells. Small.

[34] Bouremel Y., Madaan S., Lee R.M., Eames I., Wojcik A., Khaw P.T. (2017). Pursing of planar elastic pockets. J. Fluids Struct..

[35] Fluigent Fluigent ESS L-SWITCH, ESS M-SWITCH, ESS SWITCHBOARD User Manual, Version 10A. ManualZZ.

[36] Jiang S., Choudhry N. (2018). Anterior Hyaloid OCT. Ophthalmol. Retin..

[37] Khaw P.T., Shah P., Elkington A.R. (2004). Glaucoma—1: Diagnosis Primary open angle glaucoma. BMJ.

[38] Pernaut J.M., Reynolds J.R. (2000). Use of Conducting Electroactive Polymers for Drug Delivery and Sensing of Bioactive Molecules. A Redox Chemistry Approach. J. Phys. Chem. B.

[39] Dhopeshwarkar R., Crooks R.M., Hlushkou D., Tallarek U. (2008). Transient effects on microchannel electrokinetic filtering with an ion-permselective membrane. Anal. Chem..

[40] Song R., Stone H.A., Jensen K.H., Lee J. (2019). Pressure-driven flow across a hyperelastic porous membrane. J. Fluid Mech..

[41] Baskakova A., Awwad S., Jimenez J.Q., Gill H., Novikov O., Khaw P.T., Brocchini S., Zhilyakova E., Williams G.R. (2016). Electrospun formulations of acyclovir, ciprofloxacin and cyanocobalamin for ocular drug delivery. Int. J. Pharm..

[42] Krohne T.U., Liu Z., Holz F.G., Meyer C.H. (2012). Intraocular pharmacokinetics of ranibizumab following a single intravitreal injection in humans. Am. J. Ophthalmol..

[43] Xu L., Lu T., Tuomi L., Jumbe N., Lu J., Eppler S., Kuebler P., Damico-Beyer L.A., Joshi A. (2013). Pharmacokinetics of ranibizumab in patients with neovascular age-related macular degeneration: A population approach. Investig. Ophthalmol. Vis. Sci..

[44] Krohne T.U., Holz F.G., Meyer C.H. (2014). Pharmacokinetics of intravitreally administered VEGF inhibitors. Ophthalmologe.

[45] Meyer C.H., Krohne T.U., Holz F.G. (2011). Intraocular pharmacokinetics after a single intravitreal injection of 1.5 mg versus 3.0 mg of bevacizumab in humans. Retina.

[46] Zhu Q., Ziemssen F., Henke-Fahle S., Tatar O., Szurman P., Aisenbrey S., Schneiderhan-Marra N., Xu X., Grisanti S. (2008). Vitreous levels of bevacizumab and vascular endothelial growth factor-A in patients with choroidal neovascularization. Ophthalmology.

[47] Beer P.M., Wong S.J., Hammad A.M., Falk N.S., O’Malley M.R., Khan S. (2006). Vitreous levels of unbound bevacizumab and unbound vascular endothelial growth factor in two patients. Retina.

[48] Miller E., Rotea M., Rothstein J.P. (2010). Microfluidic device incorporating closed loop feedback control for uniform and tunable production of micro-droplets. Lab Chip.

[49] Goel M., Picciani R.G., Lee R.K., Bhattacharya S.K. (2010). Aqueous humor dynamics: A review. Open Ophthalmol. J..

[50] Radenbaugh P.A., Goyal A., McLaren N.C., Reed D.M., Musch D., Richards J.E., Moroi S.E. (2006). Concordance of aqueous humor flow in the morning and at night in normal humans. Investig. Ophthalmol. Vis. Sci..

[51] Otero-Millan J., Troncoso X.G., Macknik S.L., Serrano-Pedraza I., Martinez-Conde S. (2008). Saccades and microsaccades during visual fixation, exploration, and search: Foundations for a common saccadic generator. J. Vis..

[52] Meyer C.H., Lasker A.G., Robinson D.A. (1985). The upper limit of human smooth pursuit velocity. Vision Res..

[53] Erkelens C.J. (2006). Coordination of smooth pursuit and saccades. Vision Res..

[54] Foulsham T., Kingstone A., Underwood G. (2008). Turning the world around: Patterns in saccade direction vary with picture orientation. Vision Res..

[55] Higgins E., Leinenger M., Rayner K. (2014). Eye movements when viewing advertisements. Front. Psychol..

[56] Larsby B., Thell J., Möller C., Odkvist L. (1988). The effect of stimulus predictability and age on human tracking eye movements. Acta Otolaryngol..

[57] Rayner K. (1998). Eye movements in reading and information processing: 20 years of research. Psychol. Bull..

[58] Lubken R.M., de Jong A.M., Prins M.W.J. (2022). Real-Time Monitoring of Biomolecules: Dynamic Response Limits of Affinity-Based Sensors. ACS Sens..

[59] Lu X., Lozano R., Shah P. (2003). In-situ dissolution testing using different UV fiber optic probes and instruments. Dissolution Technol..

[60] Awwad S., Bouremel Y., Ibeanu N., Brocchini S.J., Khaw P.T. (2021). Artificial Eye Assembly for Studying Ocular Pharmacokinetics.

